# Antibiotic waste in a pediatric healthcare system: Wasting drugs that are in limited supply

**DOI:** 10.1017/ice.2023.118

**Published:** 2024-02

**Authors:** Lucie K. Fan, Lydia Lu, Alfred J. Fernandez, Preeti Jaggi

**Affiliations:** 1 Department of Pediatrics, Emory University School of Medicine, Atlanta, Georgia; 2 Division of Pediatric Infectious Disease, Department of Pediatrics, Children’s Healthcare of Atlanta, Atlanta, Georgia; 3 Division of Pediatric Infectious Disease, Department of Pediatrics, Emory University School of Medicine, Atlanta, Georgia

## Abstract

In a pediatric hospital system over 2 years, 58,607 doses of antibiotic were wasted, an average of 80 doses per day, including drugs in shortage nationwide. Approximately 50% of waste occurred within the first 2 days of admission or the day of discharge, with ampicillin being the most wasted drug (N = 7,789 doses).

Antibiotic waste, defined as discarded unused doses, can exacerbate drug shortages and antimicrobial resistance if broader drugs are used. It also leads to unnecessary environmental waste, which increases healthcare-associated carbon emissions without benefits to patients.^1^ Drug shortages have become more prevalent in the past several decades.^2^ In a survey of infectious diseases physicians, 78% reported needing to modify treatment due to drug shortages.^3^ Drug shortages may lead to suboptimal therapeutic options that can lengthen hospitalization^4^ and/or decrease quality of life if replaced with broader antibiotics.^5^ We quantified the antibiotics waste in our hospital system and estimated the associated financial costs and environmental waste. We also identified opportunities to decrease unnecessary waste.

## Methods

This study was conducted at Children’s Healthcare of Atlanta, a tertiary-care pediatric healthcare system comprising 3 freestanding hospitals with >600 beds. The pharmacy prints 3 batches of labels (ie, at 6:00 a.m., 12:00 p.m., and 8:00 p.m.) daily for medications due in 8–14 hours after they are printed (eg, labels printed at 6:00 a.m. for medications due between 2:00 p.m. and 8:00 p.m.). We retrospectively identified wasted antibiotic doses dispensed by the pharmacy from January 1, 2020, through December 31, 2021. Antibiotics prepared in intravenous dilutions or with weight-based dosage cannot be reused. Wasted antibiotics were defined as unused doses returned to the pharmacy, unable to be reassigned, for which a financial credit was applied to patient charges. We used an automated report to retrieve the order details of wasted antibiotics, including admission and discharge dates, weight, volume, and ordering service. The report was validated by random chart review over a 2-week period to verify doses identified. Additionally, nursing staff on one unit confirmed the antibiotics were returned due to discontinuation or patient discharge over the validation period.

We estimated the total weight and cost of waste with 2022 wholesale pricing for antibiotics, syringes, and normal saline. Since each liquid dose was prepared with saline and ≥1 disposable syringe, we assumed the saline and syringe used were wasted per liquid dose. The weight of 100 syringes is 0.866 kg^6^ with an estimated cost of $0.25 per syringe.

The following equations were used to calculate the weight and cost.

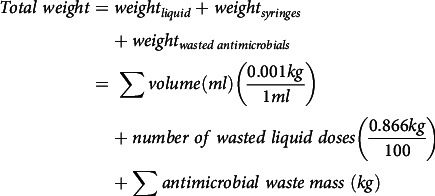




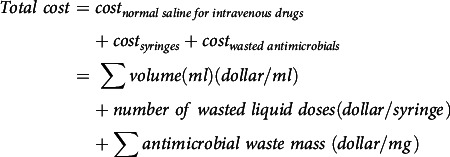




## Results

**Figure 1.**
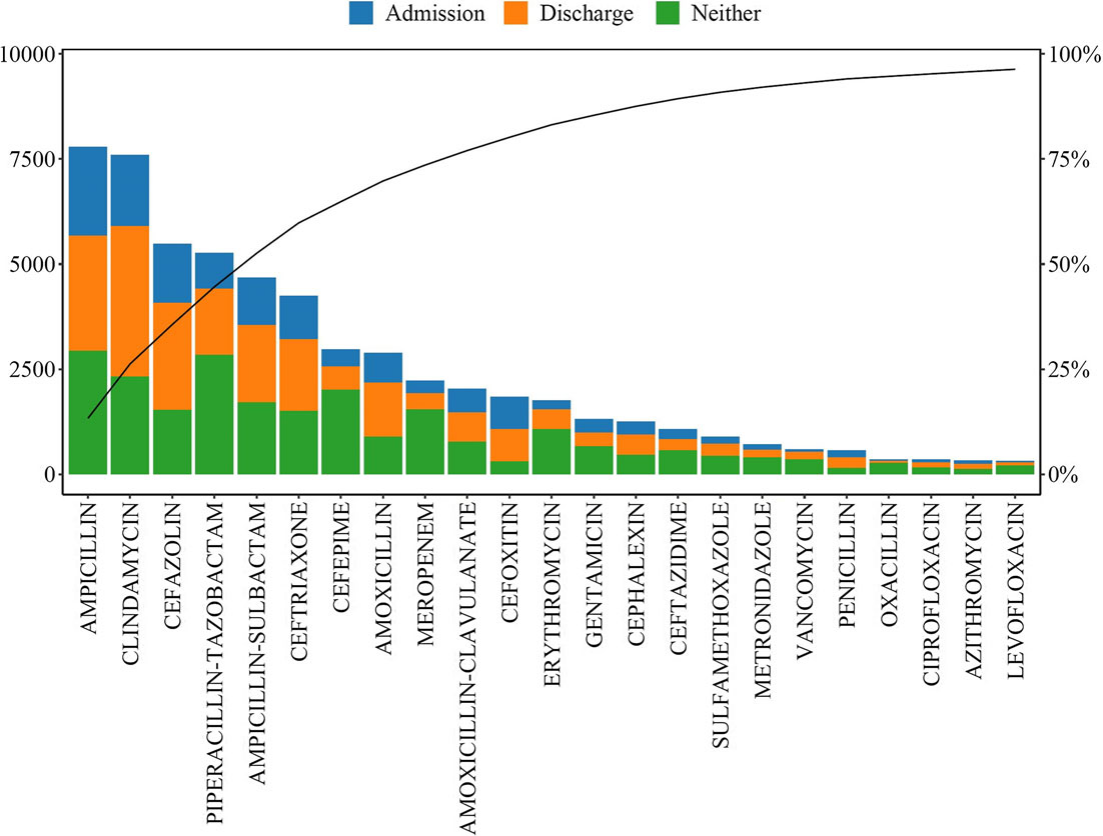
List of the 23 most wasted antibiotics over the study period. Doses wasted on “admission” include those wasted on hospital day 1 or 2 and “discharge” is on the day of discharge.

Over a 2-year period, 58,607 doses of antibiotics were wasted from 17,319 patients, an average of 80 doses per day and 169 doses per 1,000 patient days. Overall, 58,280 doses (99.4%) were either intravenous or suspension formulations (along with the same number of syringes) and the rest were tablets. Also, 13,214 doses (23%) occurred on hospital day 1 or 2 of admission, and 21,045 doses (36%) occurred on the day of patient discharge. Furthermore, 10 antibiotics accounted for 77% of the doses wasted. Ampicillin was most frequently wasted (7,789 doses, 13%), followed by clindamycin (7,597 doses, 13%) and cefazolin (5,486 doses, 9%) (Fig. 1). Medications prescribed in the general pediatrics service accounted for 24,091 doses (41%), followed by critical care (5,433 doses, 9%), hematology-oncology (4,524 doses, 8%), and surgery (3,754 doses, 6%). The weight of estimated waste was 2,507.96 kg, which included the plastic syringes, antibiotics, and carrier fluid. The cost of estimated waste was $255,503 over the 2-year period (or $127,751 annually). The cost of syringe and normal saline were $14,570 and $7,805, respectively; most of the cost was the antibiotics themselves ($233,128). The 5 most expensive antibiotic wasted per mg were tigecycline (5 doses), ceftaroline (84 doses), ceftazidime-avibactam (30 doses), ertapenem (16 doses), sulfamethoxazole-trimethoprim intravenous (163 doses). These drugs alone cost $36,541 over the 2-year period.

## Discussion

We quantified antibiotics wasted over a 2-year period, extrapolating the resulting financial and environmental impact. Approximately 80 doses are wasted daily, consistent with the large amounts of waste reported at another pediatric center.^7^ Of the 10 most wasted antibiotics, 8 were either currently in shortage or had documented shortage in the past 3 years.^8^ Although we did not estimate nonantibiotic drugs wasted, leveraging antimicrobial stewardship efforts to decrease waste on the day of discharge could lead to broader strategies preventing systemwide drug waste.

The amount of waste is high, partially avoidable, with no contribution to improving patient outcomes. Antibiotic waste resulted in >$120,000 loss annually. Additional costs should be considered, including staff time preparing, managing, and accounting for wasted drugs, and costs associated with suboptimal treatments due to shortages exacerbated by waste. High-priority targets to use antibiotic resources wisely include empiric optimization to minimize drug changes within 24 hours of admission and planning around the time of discharge. Antimicrobial stewardship teams are often tasked with creating protocols to optimize empiric treatment choices. Most empiric use should be limited to 36–48 hours pending blood cultures with automatic stop times and careful monitoring. Additionally, medication is wasted if the pharmacy receives a discontinuation notification during morning rounds after it was prepared. Therefore, high-turnover services, such as general pediatrics, can decrease waste through judicious ordering and early discharge planning.^7^ At least 36% of drugs were wasted on the day of discharge, likely preventable if discharge planning included stop times. Facilitating communication with the pharmacy could be considered in these services. In a multicenter retrospective study, pharmacy predischarge huddle with planned antibiotic course was shown to help decrease waste.^9^

Given these findings, we instituted automatic stop times for situations such as febrile neonates who received 36 hours of antibiotics with pending blood cultures and patients treated for otitis media. The prospective audit-and-feedback strategy of our antimicrobial stewardship team may unintentionally lead to more antibiotic changes and waste than would be seen in a system primarily utilizing antimicrobial restriction. Therefore, we are considering incorporating automatic stop times and/or prior authorization for broad spectrum antibiotics. We also organized an educational campaign for providers to urge early discharge planning (eg, changing to oral therapy sooner). In the neonatal, cardiac, and pediatric intensive care units, we encouraged predischarge huddles with charge nurses. We are currently re-examining our pharmacy batch times as well.

The healthcare sector accounts for 8.5% of carbon emissions, and most are considered “indirect.” An example of this is the purchase, use, and disposal of products from suppliers.^1^ We recognized that decreasing drug waste would decrease the associated emissions associated with the lifecycle of those drugs, and this is a measurable outcome that hospitals can undertake to remove unnecessary environmental impact.

This study had several limitations. It was a single-center study, though high rates of anti-infective waste have been noted elsewhere.^7^ Not all drugs credited were due to the lack of appropriate stop times. Drugs could have been lost or dropped by providers. Our findings are also not generalizable to all, as antibiotic waste likely occurs more often in pediatric settings due to weight-based dosing and use of suspension, which cannot be restocked. We noted that the wholesale pricing fluctuates so the cost might be time dependent.

In conclusion, systematic changes to decrease antibiotic waste in pediatric hospitals are needed. Strategies focusing on discharge planning for high-turnover services can have the greatest initial impact on reducing antibiotic waste.
